# ASB9 interacts with ubiquitous mitochondrial creatine kinase and inhibits mitochondrial function

**DOI:** 10.1186/1741-7007-8-23

**Published:** 2010-03-19

**Authors:** Sanghoon Kwon, Dongbum Kim, Jae Won Rhee, Jeong-A Park, Dae-Won Kim, Doo-Sik Kim, Younghee Lee, Hyung-Joo Kwon

**Affiliations:** 1 Department of Microbiology, College of Medicine, Hallym University, Gangwon-do, Republic of Korea; 2 Center for Medical Science Research, College of Medicine, Hallym University, Gangwon-do, Republic of Korea; 3 Department of Biochemistry, College of Natural Sciences, Chungbuk National University, Chungbuk, Republic of Korea; 4 Department of Biochemistry, College of Science, Yonsei University, Seoul, Republic of Korea

## Abstract

**Background:**

The ankyrin repeat and suppressor of cytokine signalling (SOCS) box proteins (Asbs) are a large protein family implicated in diverse biological processes including regulation of proliferation and differentiation. The SOCS box of Asb proteins is important in a ubiquitination-mediated proteolysis pathway. Here, we aimed to evaluate expression and function of human Asb-9 (ASB9).

**Results:**

We found that a variant of ASB9 that lacks the SOCS box (ASB9ΔSOCS) was naturally detected in human cell lines but not in peripheral blood mononuclear cells or normal hepatocytes. We also identified ubiquitous mitochondrial creatine kinase (uMtCK) as a new target of ASB9 in human embryonic kidney 293 (HEK293) cells. The ankyrin repeat domains of ASB9 can associate with the substrate binding site of uMtCK in a SOCS box-independent manner. The overexpression of ASB9, but not ASB9ΔSOCS, induces ubiquitination of uMtCK. ASB9 and ASB9ΔSOCS can interact and colocalise with uMtCK in the mitochondria. However, only expression of ASB9 induced abnormal mitochondrial structure and a decrease of mitochondrial membrane potential. Furthermore, the creatine kinase activities and cell growth were significantly reduced by ASB9 but not by ASB9ΔSOCS.

**Conclusions:**

ASB9 interacts with the creatine kinase system and negatively regulates cell growth. The differential expression and function of ASB9 and ASB9ΔSOCS may be a key factor in the growth of human cell lines and primary cells.

## Background

The largest family of suppressor of cytokine signalling (SOCS) box-containing superfamily proteins are the ankyrin repeat and SOCS box proteins (Asbs; ASBs in humans). Although 18 members of the Asb family have been identified in mice and humans, the function of Asbs has not been clearly defined. The Asbs have two functional domains, a SOCS box and a variable number of N-terminal ankyrin (ANK) repeats [1]. The SOCS box of Asb proteins has two subdomains: a BC box and a Cul2/Cul5 box. Highly conserved amino acid sequences of the BC box and the Cul5 box, which are essential for ensuring that the interaction with elongins B/C and Cullin 5-Rbx2 forms E3 ubiquitin (Ub) ligase complexes, are important in a ubiquitination-mediated proteolysis pathway [2-6]. While SOCS family members use the SH2 domain to recruit substrates, the ANK repeat regions of Asb family members serve as specific protein-protein interaction platforms to recruit target substrates in different biological processes [1]. Asb-2 targets the actin-binding proteins filaments A and B for proteasomal degradation. Heuze *et al*. have shown that Asb-2 may regulate haematopoietic cell differentiation by modulating the cell-spreading process [4]. ANK repeats of Asb-3 interact with the C-terminus of tumour necrosis factor (TNF)-R2 and act as a negative regulator of the TNF-R2-mediated cellular response [3]. The ANK repeat of Asb-4 interacts with the factor-inhibiting HIF1α (FIH); it is also a hydroxylation substrate of FIH and promotes vascular differentiation [7]. Asb-15 reportedly regulates protein synthesis in skeletal muscle and alters the differentiation of mouse myoblast and the phosphorylation of mitogen-activated protein kinase and Akt [8,9].

Creatine kinases (CKs) are a large family of isoenzymes involved in high-energy phosphate metabolism. Five isoforms of CK have been identified in mammals: three in cytosol, namely CKB (brain type), CKM (muscle type) and CKMB (mixed type); and two in mitochondria, namely ubiquitous mitochondrial CK (uMtCK) and sarcomeric MtCK (sMtCK) [10,11]. CKs regulate levels of ATP in subcellular compartments, where they provide ATP molecules at sites of fluctuating energy demand of very specialised cells, such as muscle fibres, neurons, or sperm cells, by the transfer of phosphates between creatine and adenine nucleotides and immediate regeneration of ATP. The CKs are also necessary for maintaining the normal function of many other cells, including kidney, placenta, pancreas, thymus, thyroid, and intestinal epithelial cells, as well as endothelial cells, cartilage and bone cells, macrophages, and blood platelets, possibly by a continuous delivery of high-energy phosphates to the site of ATP utilisation [10-14].

Abnormal expression and activity of CKs have been shown in tumours and diseases [11,15,16]. The cytosolic CKB is induced in a variety of tumours, including neuroblastoma, small cell lung carcinoma, colon adenocarcinoma, and breast carcinoma. Because the tumour suppressor p53 is involved in the repression of CKB transcription, CKB may be increased in tumours with mutations in p53 alleles [17]. MtCK is known as a primary target of oxidative and radical-induced molecular damage; and the impairment of MtCK has been reported in ischaemia, cardiomyopathy, and neurodegenerative disorders [18,19]. Upregulation of MtCK has also been shown in creatine-depleted muscles and in patients with mitochondrial cytopathies, which may explain the mechanism of compensating for the functional impairment of the energy state control. Overexpression of MtCK in tumours has also been reported [20-22]. Hence, CKs and creatine analogues are considered a new target of chemotherapeutics for cancer.

A recent report shows that ASB9 interacts with creatine kinase B CKB in human embryonic kidney 293 (HEK293) cells, indicating that human Asb-9 (ASB9) mediates the ubiquitination and proteasomal degradation of CKB in cells [23]. ASB9 was isolated as a potential biomarker for breast cancer [24]. In this study, we report that a variant of ASB9 that lacks a SOCS box was naturally detected in human cell lines but not in human primary cells. We also demonstrate that ASB9 interacts with uMtCK and induces malfunction of mitochondria, leading to negative regulation of cell growth.

## Results

### Identification of a variant form of ASB9 in human cell lines

**Figure 1:**
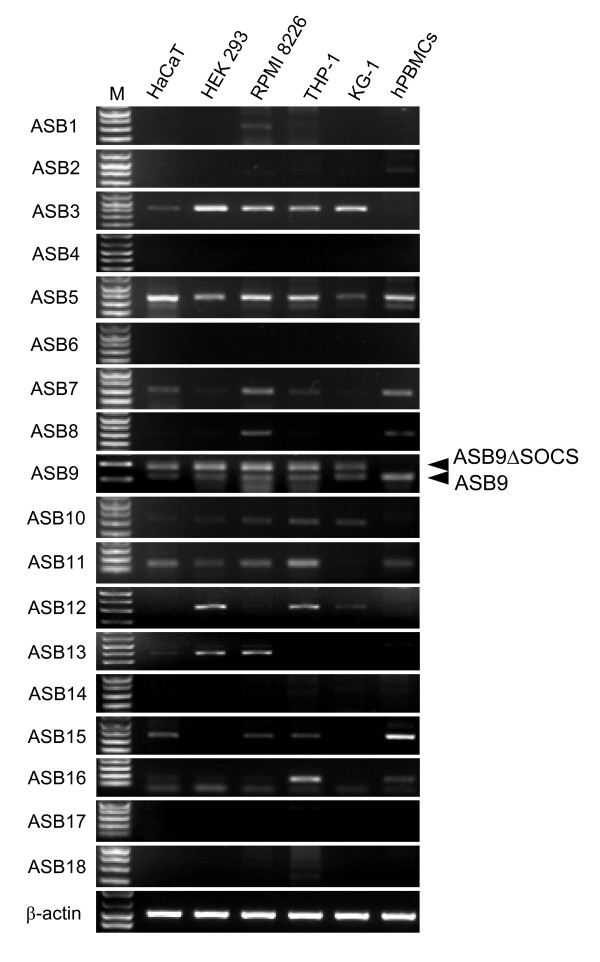
**Expression of 18 members of the ankyrin repeat and suppressor of cytokine signalling (SOCS) box protein (Asb) family in human cell lines and human peripheral blood mononuclear cells (hPBMCs)**. The human keratinocyte (HaCaT), human embryonic kidney 293 (HEK293), RPMI 8226, THP-1, KG-1, and human peripheral blood mononuclear cells were cultured for 1 day before total RNA isolation. The total RNAs were extracted and reverse transcribed to generate cDNAs. A total of 1 μl of the cDNA mixture was subjected to the standard PCR reaction for 25 cycles with the primer sets as described in Table 2. The β-actin expression level was used as a control. The letter M denotes a standard DNA marker.

**Figure 2:**
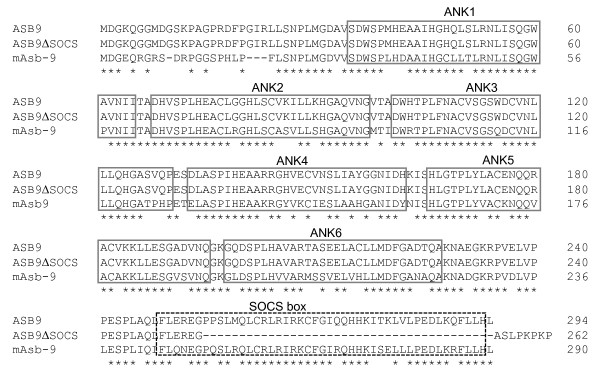
**Sequences of human ankyrin repeat and suppressor of cytokine signalling (SOCS) box protein 9 (ASB9), ASB9ΔSOCS and mAsb-9**. CLUSTAL alignment [39] of ASB9, ASB9ΔSOCS, and mAsb-9; the location of the N-terminal ankyrin (ANK) repeats and the SOCS box motif are shown.

We used reverse transcription polymerase chain reaction (RT-PCR) to analyse the expression pattern of 18 members of the ASB family in human cell lines and human peripheral blood mononuclear cells (hPBMCs). As shown in Figure 1, two bands of ASB9 PCR products (115 bp of expected size and 175 bp of longer size) were detected in human cell lines. However, hPBMC expressed only one band of ASB9. Nucleotide sequence analysis of the two cDNA bands shows that the shorter band represents the ASB9 gene (GenBank accession number, NM_001031739). Interestingly, the longer PCR product represented a splicing variant of ASB9 (GenBank accession number, NM_024087). We amplified cDNA sequences that encoded full-length open reading frames of ASB9 and the variant from HEK293 cells by RT-PCR. When we deduced the amino acid sequences from the cDNAs, we found the splicing variant encodes a smaller protein with a deletion of the SOCS box domain from ASB9, which we named ASB9ΔSOCS (Figure 2). ASB9 and ASB9ΔSOCS have six ankyrin repeats.

**Figure 3:**
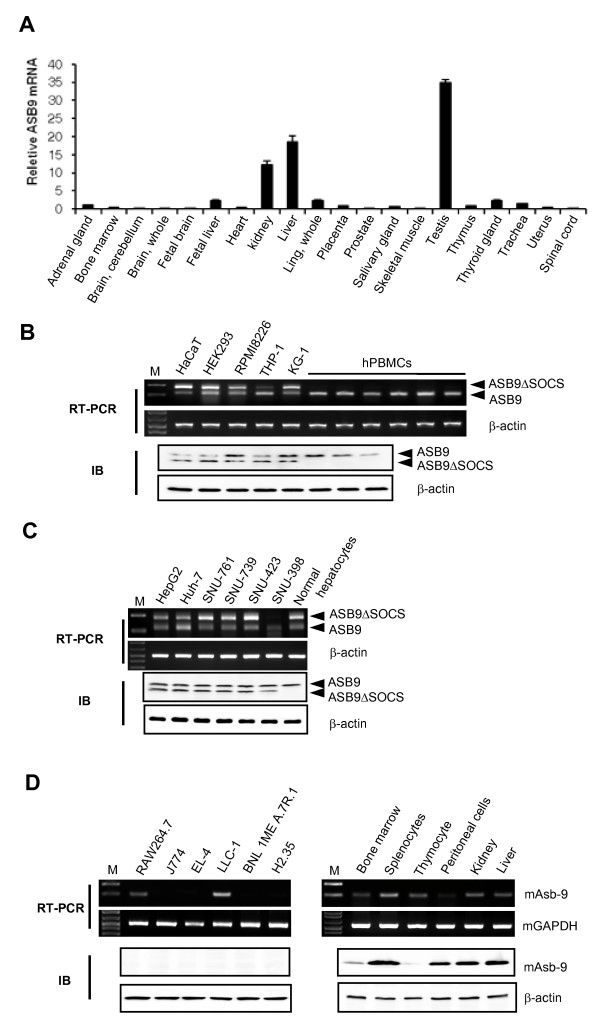
**Expression patterns of human ankyrin repeat and suppressor of cytokine signalling (SOCS) box protein 9 (ASB9) and ASB9ΔSOCS**. **(a) **The expression level of ASB9 mRNA was analysed by real-time PCR using the cDNAs from indicated human tissues. The mRNA levels were normalised using glyceraldehyde 3-phosphate dehydrogenase (GAPDH) as an internal control, and relative expression was determined by dividing all normalised values within a data set by the normalised arbitrary units of the control. **(b) **Expression of ASB9 in human cell lines and human peripheral blood mononuclear cells (hPBMCs). **(c) **Expression of ASB9 in human hepatoma cell lines and normal human hepatocytes. The expression of ASB9 mRNA and protein was analysed by means of reverse transcription (RT)-PCR and immunoblotting, respectively. The β-actin expression level was used as a control. **(d) **Expression of mAsb-9 in mouse cell lines and lymphoid tissues. The GAPDH expression level was used as an mRNA control. The expression of mAsb-9 protein in the mouse cell lines and primary cells was analysed by immunoblotting with anti-ASB9 antibody. The β-actin expression level was used as a protein control.

To examine the tissue distribution of the ASB9 expression, we performed real-time PCR analysis on cDNAs prepared from human adult tissues. The ASB9 mRNA was expressed predominantly in the testes, kidney, and liver (Figure 3a). The ASB9 and ASB9ΔSOCS expression was detected in human cell lines such as the leukaemia and hepatoma cell lines at the mRNA and protein levels (Figure 3b, c). Interestingly, the ASB9ΔSOCS expression was undetectable in the hPBMCs from diverse blood samples and human normal hepatocytes (Figure 3b, c). However, a mouse Asb-9 without SOCS box (mAsb-9ΔSOCS) was not detected in the mouse cell lines and tissues (Figures 2 and 3d).

### Association of ASB9 with CKs and ubiquitin ligase complexes

**Figure 4:**
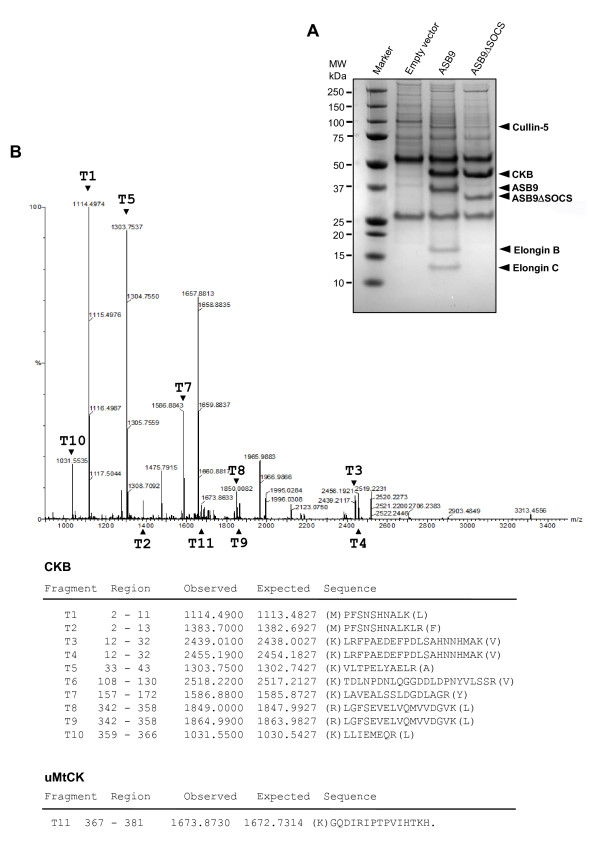
**Identification of human ankyrin repeat and suppressor of cytokine signalling (SOCS) box protein 9 (ASB9) binding proteins**. **(a) **Cell lysates were prepared from human embryonic kidney 293 (HEK293) cells that were stably transfected with the indicated expression vectors; immunoprecipitates were then generated by using an antibody to Myc epitope, after which they were resolved by SDS-PAGE and stained with Coomassie brilliant blue G-250. **(b) **The protein bands coimmunoprecipitated with ASB9 were digested in gel with trypsin, and the samples were analysed by electrospray ionization-time of flight mass spectrometry/mass spectrometry (ESI-TOF MS/MS). MS/MS analyses of the mass peaks (arrow) obtained from the 45-kDa band reveal the peptide spectra of CKB and ubiquitous mitochondrial creatine kinase (uMtCK).

Because SOCS box-containing proteins associate with substrate proteins for degradation, immunocomplexing assays were performed to identify the ASB9 interacting proteins (Figure 4a). An anti-Myc antibody was used to generate immunocomplexes from HEK293 cells that stably express null, Myc-tagged ASB9 or ASB9ΔSOCS proteins. The proteomic analysis suggests that the main protein associated with ASB9 and ASB9ΔSOCS is CKB. Peptide fragments detected by mass spectroscopy show 27% sequence coverage of the total amino acids of CKB (Figure 4b and Table 1); this result confirms that ASB9 interacts with endogenous CKB in a SOCS box-independent manner. Note also that ASB9, but not ASB9ΔSOCS, coimmunoprecipitated with Cullin 5, elongin B and elongin C (Figure 4a and Table 1). This behaviour indicates that ASB9 interacts with endogenous Cullin 5 and elongin B/C through its SOCS box, which is consistent with a recent report [23]. Additionally, we isolated another peptide sequence from uMtCK, even though the sequence contained only 15 amino acids and covered only 4% of the entire uMtCK sequence (Figure 4b and Table 1). Importantly, the sequence corresponds to the C-terminal region (amino acids 367 to 381) of uMtCK where the sequence shows only 27% identity with CKB (Figure 4b).

**Table 1:** Proteins identified by mass spectrometry

Identified proteins	**GenBank accession no**.	Molecular weight (kDa)	Matched peptides	Sequence coverage
CKB	AAC31758	43	106	27%
uMtCK	NP066270	46	15	4%
Cullin 5	NP003469	91	86	11%
Elongin B	AAC08452	18	32	44%
Elongin C	NP005639	10	20	17%

The protein bands coimmunoprecipitated with human ankyrin repeat and suppressor of cytokine signalling (SOCS) box protein (ASB9) (Figure 4a) were digested in gel with trypsin, and the samples were analysed by electrospray ionization-time of flight mass spectrometry/mass spectrometry (ESI-TOF MS/MS).

CKB = creatine kinase B; uMtCK = ubiquitous mitochondrial CK.

**Figure 5:**
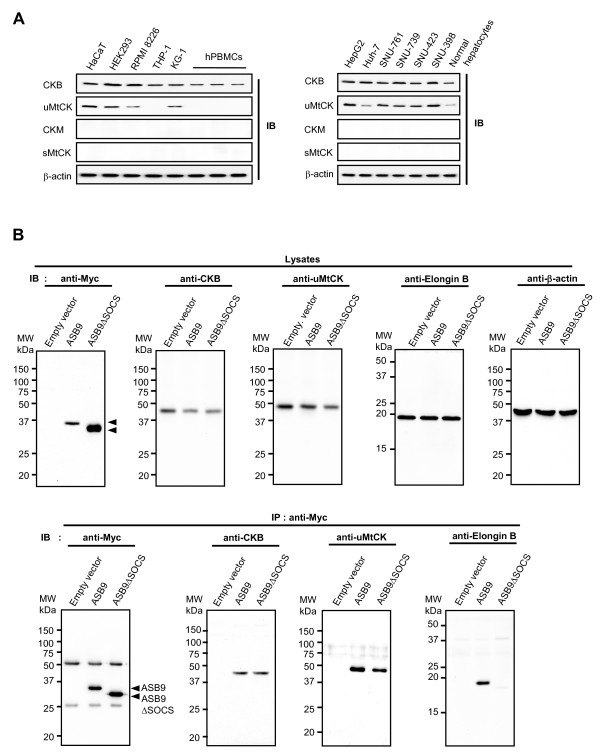
**Creatine kinase (CK)-binding specificities of human ankyrin repeat and suppressor of cytokine signalling (SOCS) box protein 9 (ASB9)**. **(a) **Expression of CKs in human normal hepatocytes human hepatoma cell lines and normal human hepatocytes. Whole lysates were subjected to immunoblotting (IB) with anti-CKB, anti-ubiquitous mitochondrial CK (uMtCK), and anti-CKM, and anti-sarcomeric MtCK (sMtCK) antibodies. The β-actin expression level was used as a control. **(b) **Association of ASB9 with CKB and uMtCK. The human embryonic kidney 293 calls (HEK293) cells were stably transfected with empty, Myc-tagged ASB9 and ASB9ΔSOCS expression vectors. Whole lysates or immunoprecipitates obtained with the anti-Myc antibody were subjected to immunoblotting (IB) analysis with anti-Myc, anti-CKB, anti-uMtCK, and anti-elongin B antibodies.

**Figure 6:**
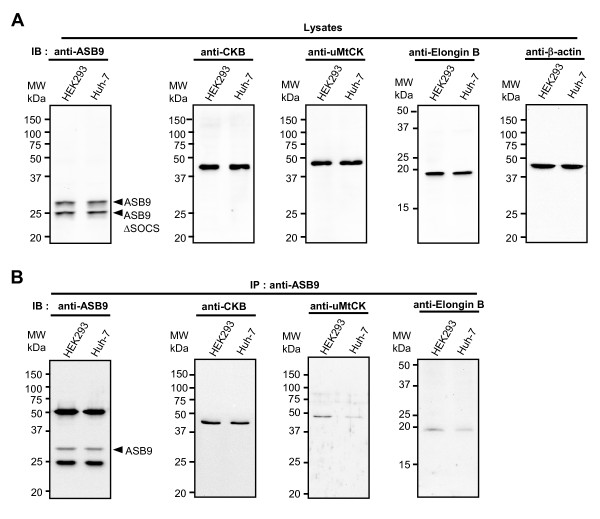
**Expression and creatine kinase (CK)-binding specificities of endogenous human ankyrin repeat and suppressor of cytokine signalling (SOCS) box protein 9 (ASB9)**. **(a) **Expression of endogenous ASB9 and ASB9ΔSOCS. Cell lysates of human embryonic kidney 293 (HEK293) cells and Huh-7 cells were used to show endogenous expression of ASB9 and ASB9ΔSOCS. Endogenous expression of CKB, mitochondrial CK (MtCK), elongin B was also confirmed by immunoblotting. **(b) **CK-binding activity of endogenous ASB9. The immunoprecipitates obtained with the anti-ASB9 antibody were analysed by immunoblotting (IB) with anti-ASB9, anti-CKB, anti-uMtCK, and anti-elongin B antibodies.

Given that the isoforms of CK are highly homologous, we decided to check the CK-binding specificity of ASB9 in the HEK293 cells. First, the expression of endogenous CKB and uMtCK was identified by immunoblotting analysis. In contrast, the expression of CKM and sMtCK was not observed (Figure 5a). Next, the interaction specificity between ASB9 and CKs was determined by immunocomplexing assays. In HEK293 cells that stably express Myc-tagged ASB9 and ASB9ΔSOCS, endogenous CKB and uMtCK coimmunoprecipitated with ASB9 and ASB9ΔSOCS (Figure 5b). In agreement with mass spectroscopy analysis (Figure 4), ASB9, but not ASB9ΔSOCS, coimmunoprecipitated with elongin B. To further confirm endogenous interaction of these proteins, immunocomplexes were obtained using anti-ASB9 antibody and cell lysates from two cell lines HEK293 and Huh-7 and analysed by immunoblotting (Figure 6). As expected, all of the proteins CKB, uMtCK and elongin B were identified in the immunoprotein complexes. Others have previously shown interaction of ASB9 with CKB [23], but the interaction with uMtCK is a new finding. We therefore decided to check the interaction of ASB9 with uMtCK in detail.

### Interaction of the ANK repeats of ASB9 with the substrate binding site of uMtCK

**Figure 7:**
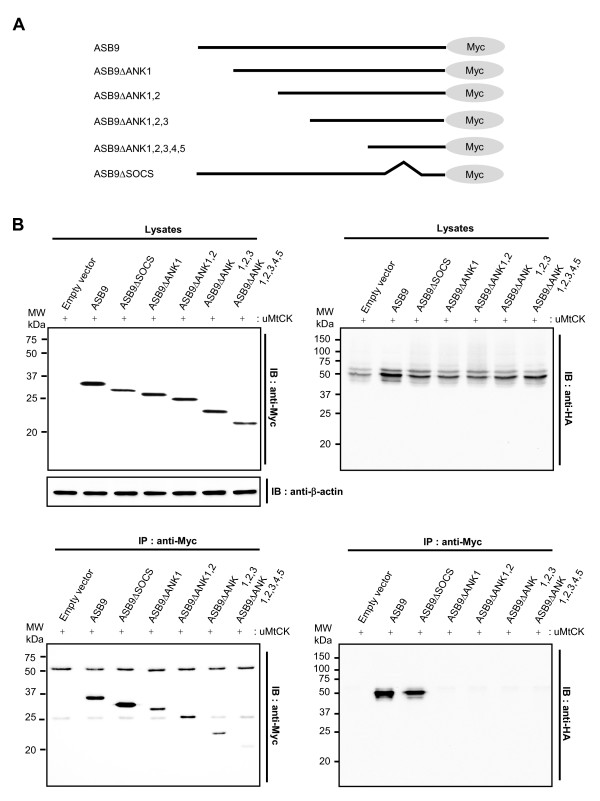
**Interaction of human ankyrin repeat and suppressor of cytokine signalling (SOCS) box protein (ASB9) with ubiquitous mitochondrial creatine kinase (uMtCK)**. **(a) **Schematic diagram of the ASB9 and a series of ASB9 N-terminal ankyrin (ANK) deletion mutants. **(b) **ANK repeats of ASB9 are required for uMtCK interaction. Myc-tagged ASB9 and a series of ANK deletion mutants were transiently cotransfected with haemagglutinin (HA)-tagged uMtCK into human embryonic kidney 293 (HEK293) cells. Cell lysates and an immunocomplex of the anti-Myc antibody were analysed by immunoblotting with anti-Myc or anti-HA antibodies.

To clarify the interaction between ASB9 and uMtCK, we used a coimmunocomplexing assay and mapped the regions of ASB9 and uMtCK involved in the protein-protein interaction. To determine which region of ASB9 is essential for binding to uMtCK, a series of Myc-tagged ASB9 deletion constructs that encode various numbers of ANK repeat motifs was made (Figure 7a) and cotransfected with haemagglutinin (HA)-tagged uMtCK constructs into HEK293 cells. The coimmunocomplexing assay shows that ASB9 interacted with uMtCK in a SOCS box-independent manner (Figure 7b, lower right). However, the deletion of ANK repeat 1 (ASB9ΔANK1) abrogated its interaction with uMtCK (Figure 7b, lower right). Further deletion of the remaining ANK repeats showed the same consequences as the deletion of the first ANK repeat (ASB9ΔANK1), suggesting that ANK repeat 1 is essential for ASB9 and uMtCK interactions.

**Figure 8:**
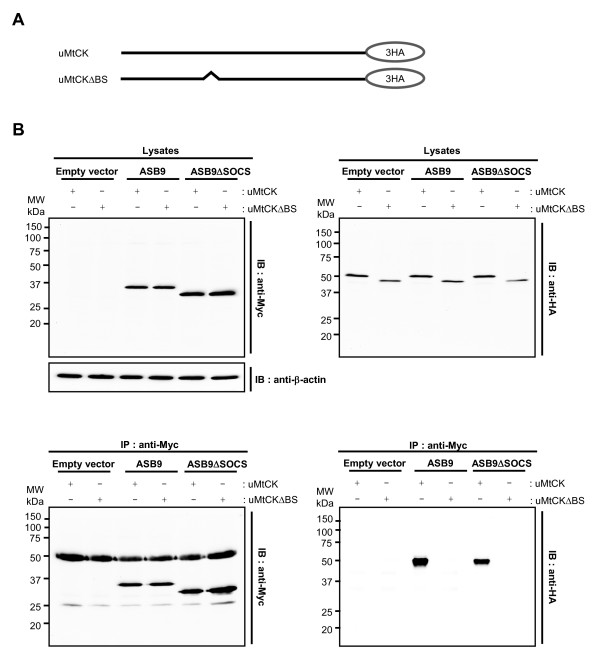
**Interaction of human ankyrin repeat and suppressor of cytokine signalling (SOCS) box protein 9 (ASB9) with substrate binding site of ubiquitous mitochondrial creatine kinase (uMtCK)**. **(a) **Schematic diagram of the uMtCK and an uMtCK substrate binding site (BS) deletion mutant. **(b) **A substrate binding site of uMtCK is required for ASB9 interaction. Haemagglutinin (HA)-tagged uMtCK or uMtCKΔBS were transiently cotransfected with Myc-tagged ASB9 or ASB9ΔSOCS into human embryonic kidney 293 (HEK293) cells, and immunocomplexing assays were performed with an anti-Myc antibody. Cell lysates and immunoprecipitates were analysed by immunoblotting with anti-Myc or anti-HA antibodies.

CKs are divided into three subdomains: a substrate binding site, an N-terminal catalytic domain, and a C-terminal catalytic domain. The substrate binding site is expected to recruit interacting proteins [25,26]. To determine whether the substrate binding site of uMtCK is essential for its interaction with ASB9, we constructed a HA-tagged substrate binding site (BS) deletion mutant (uMtCKΔBS) (Figure 8a) and cotransfected it with Myc-tagged ASB9 constructs into HEK293 cells (Figure 8b). Both ASB9 and ASB9ΔSOCS coimmunoprecipitated with uMtCK but not with uMtCKΔBS (Figure 8b, lower right). These results indicate that the ANK repeat domains of ASB9 can associate with the substrate binding site of uMtCK in a SOCS box-independent manner.

### The ASB9-induced ubiquitination and degradation of uMtCK

**Figure 9:**
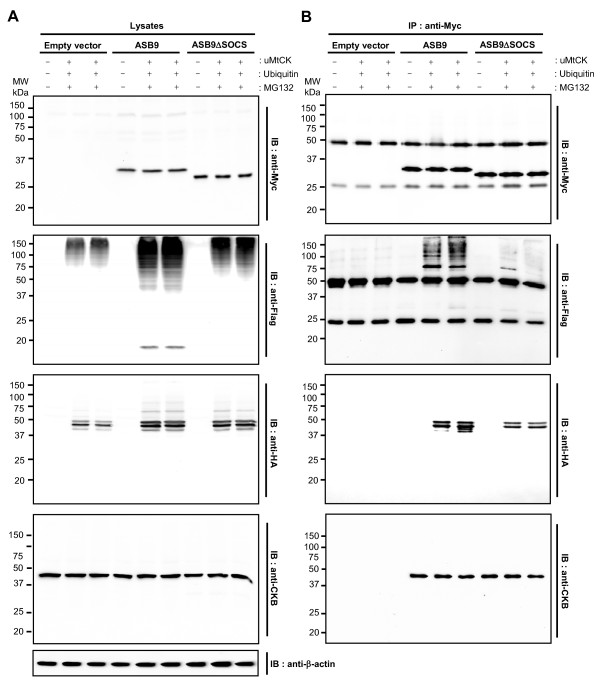
**Suppressor of cytokine signalling (SOCS) box-dependent ubiquitination of creatine kinase B (CKB) and ubiquitous mitochondrial CK (uMtCK) by human ankyrin repeat and SOCS box protein 9 (ASB9)**. Haemagglutinin (HA)-tagged uMtCK was transiently cotransfected with Flag-ubiquitin (Ub) into HEK293 cells that stably express null (empty vector), Myc-tagged ASB9 or ASB9ΔSOCS. The cells were pretreated with MG132 for 6 h prior to the cell harvest when indicated. Cell lysates **(a) **and the anti-Myc antibody immunocomplexes **(b) **were blotted with anti-Myc, anti-Flag, anti-HA or anti-CKB antibodies.

Because ASB9 assembles with an ubiquitin ligase complex (Figure 4), ubiquitination of ASB9-associated proteins (CKB and uMtCK) was evaluated by *in vivo *ubiquitination assays. When uMtCK and ubiquitin were introduced, high-molecular-weight bands representing the ubiquitination of the associated proteins were clearly detected in the ASB9-expressing HEK293 cells (Figure 9a). The polyubiquitinated proteins accumulated in the presence of MG-132 (Figure 9b). In contrast, significant ubiquitination of the ASB9-interacting proteins was not observed in the cells that express ASB9ΔSOCS, probably because ASB9ΔSOCS can't assemble with an Ub ligase complex (Figure 9).

**Figure 10:**
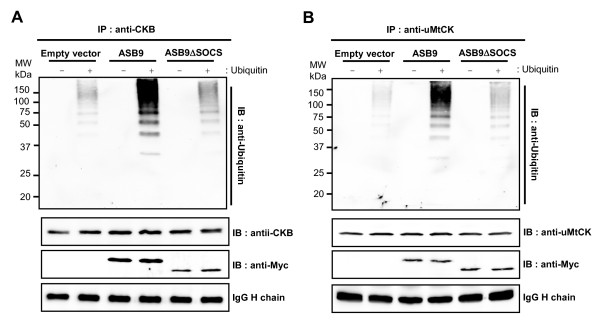
***In vitro *ubiquitination of creatine kinase B (CKB) and ubiquitous mitochondrial CK (uMtCK) by human ankyrin repeat and suppressor of cytokine signalling (SOCS) box protein 9 (ASB9)**. Whole cell lysates were prepared from human embryonic kidney 293 (HEK293) cells that stably express null (empty vector), Myc-tagged ASB9 or ASB9ΔSOCS and then immunoprecipitated with anti-CKB antibody **(a) **or anti-uMtCK antibody **(b)**. The immunocomplexes were analysed by means of *in vitro *ubiquitination assay with or without ubiquitin. The reaction mixtures were then separated by SDS-PAGE followed by immunoblotting analysis with anti-ubiquitin, anti-CKB, or anti-Myc antibodies. An immunoglobulin heavy chain is shown as an antibody amount control.

To confirm ASB9-dependent CK ubiquitination, we performed an *in vitro *ubiquitination assay (Figure 10). The lysates of null, Myc-tagged ASB9 or ASB9ΔSOCS expressing cells were immunoprecipitated with anti-CKB (Figure 10a) or anti-uMtCK antibodies (Figure 10b), and the ubiquitination of CKB and uMtCK was monitored. Polyubiquitination was not observed in the absence of ubiquitin. With ubiquitin, the polyubiquitination of CKB or uMtCK proteins was clearly detected in the presence of ASB9, and only weak polyubiquitination was seen in ASB9ΔSOCS or the empty vector control, probably because of the endogenous ASB9 activity (Figure 10). There were protein bands that migrate at a molecular weight less than CKB or uMtCK alone probably because of partial protein degradation. Thus, the results strongly indicate that ASB9 is involved in the recruitment, ubiquitination, and degradation of CKB and uMtCK through the SOCS box.

**Figure 11:**
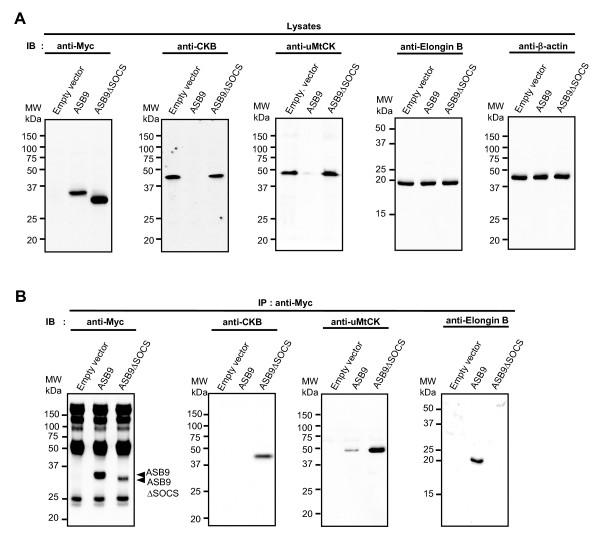
**Degradation of creatine kinase B (CKB) and ubiquitous mitochondrial CK (uMtCK) by human ankyrin repeat and suppressor of cytokine signalling (SOCS) box protein 9 (ASB9) after long-term culture**. **(a) **After culture of 10 passages of human embryonic kidney 293 (HEK293) cells stably transfected with Myc-tagged ASB9 and ASB9ΔSOCS expression vectors, cell lysates were immunoblotted with anti-Myc, anti-CKB, anti-uMtCK, and anti-elongin B antibodies. β-actin was used as a protein amount control. **(b) **The lysates obtained from HEK293 cells stably transfected with Myc-tagged ASB9 and ASB9ΔSOCS expression vectors were immunoprecipitated with an anti-Myc antibody and subjected to immunoblotting with anti-Myc, anti-CKB, anti-uMtCK, and anti-elongin B antibodies.

To investigate the long-term effect of ASB9 expression *in vivo*, we analysed the expression of CKB and uMtCK after culture of stable cell lines for 10 passages. Immunoblotting analysis with cell lysates and anti-Myc immunoprecipitates reveal that the protein levels of endogenous CKB and uMtCK were dramatically reduced in the HEK293 cells that stably express ASB9 but not in ASB9ΔSOCS (Figure 11). These results suggest that the ubiquitination and degradation by ASB9 do affect the protein levels of target proteins in the cells.

### Colocalisation of ASB9 and uMtCK in the mitochondria and influence of ASB9 expression on the mitochondrial membrane potential

**Figure 12:**
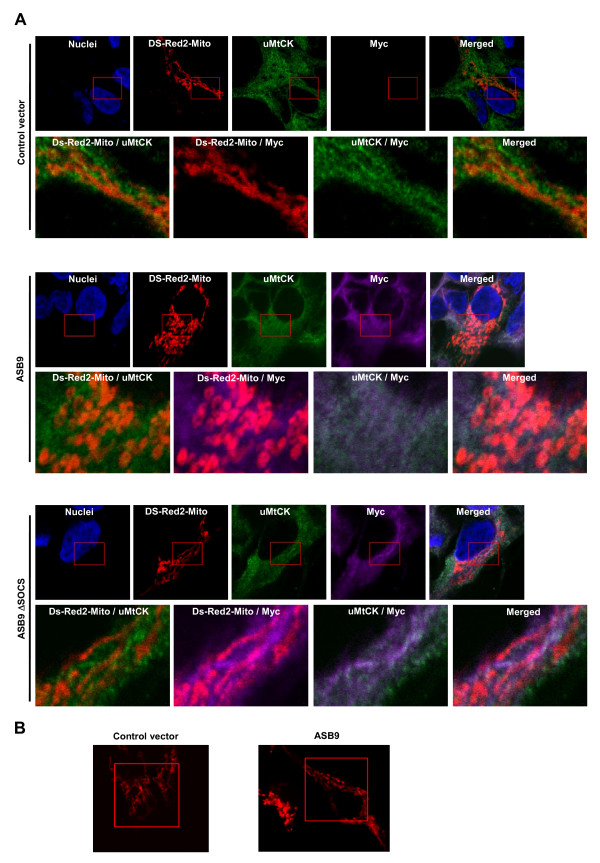
**Colocalisation of human ankyrin repeat and suppressor of cytokine signalling (SOCS) box protein 9 (ASB9) and ubiquitous mitochondrial creatine kinase (uMtCK) in mitochondria**. The pDSRed2-Mito vector was transfected into human embryonic kidney 293 (HEK293) cells that stably express null (empty vector), Myc-tagged ASB9 or ASB9ΔSOCS. After 24 h incubation, the cells were fixed with 4% paraformaldehyde. **(a) **A confocal laser scanning microscopy was used to visualise the location of individual proteins with the aid of the mouse anti-Myc antibody (for ASB9, purple) and the goat anti-uMtCK antibody (green), respectively. DSRed2-Mito was expressed in mitochondria as a red colour. Cells were stained with Hoechst no. 33258 to visualise the nuclei (blue colour). **(b) **A confocal laser scanning microscope was used to visualise the structure of mitochondria with the aid of DSRed2-Mito. Three-dimensional movies of the figures are available as Additional files 1 and 2.

Given that endogenous uMtCK can interact with ASB9 (Figure 4), we evaluated whether ASB9 colocalises with endogenous uMtCK in the mitochondria. The cellular localisation of protein was analysed by immunofluorescence staining and confocal imaging. The expression of DSRed2-Mito was used as a mitochondrial marker. Colocalisation of DSRed2-Mito and uMtCK in mitochondria was observed as expected (Figure 12a). Furthermore, ASB9 was detected in the cytosol, which also includes mitochondria, though no ASB9 was detected in the nucleus. Moreover, uMtCK and either ASB9 or ASB9ΔSOCS colocalised in the mitochondria; this colocalisation is in agreement with the result of *in vitro *association of uMtCK with ASB9 that occurs in a SOCS box-independent manner (Figures 4, 5, 6, 7, 8). Interestingly, an abnormal mitochondrial structure was observed in the ASB9-expressing HEK293 cells (Figure 12a, middle panel). To investigate mitochondrial structure in the cells in detail, we compared the architecture of mitochondria with the aid of DSRed2-Mito and three-dimensional analysis of confocal data and found rounding and swelling of mitochondria in ASB9 expressing cells (Figure 12b and Additional files 1 and 2). These results revealed that the ASB9 interacts and colocalises with uMtCK in the mitochondria, leading to structural organisation defects, which occur in a SOCS box-dependent manner.

**Figure 13:**
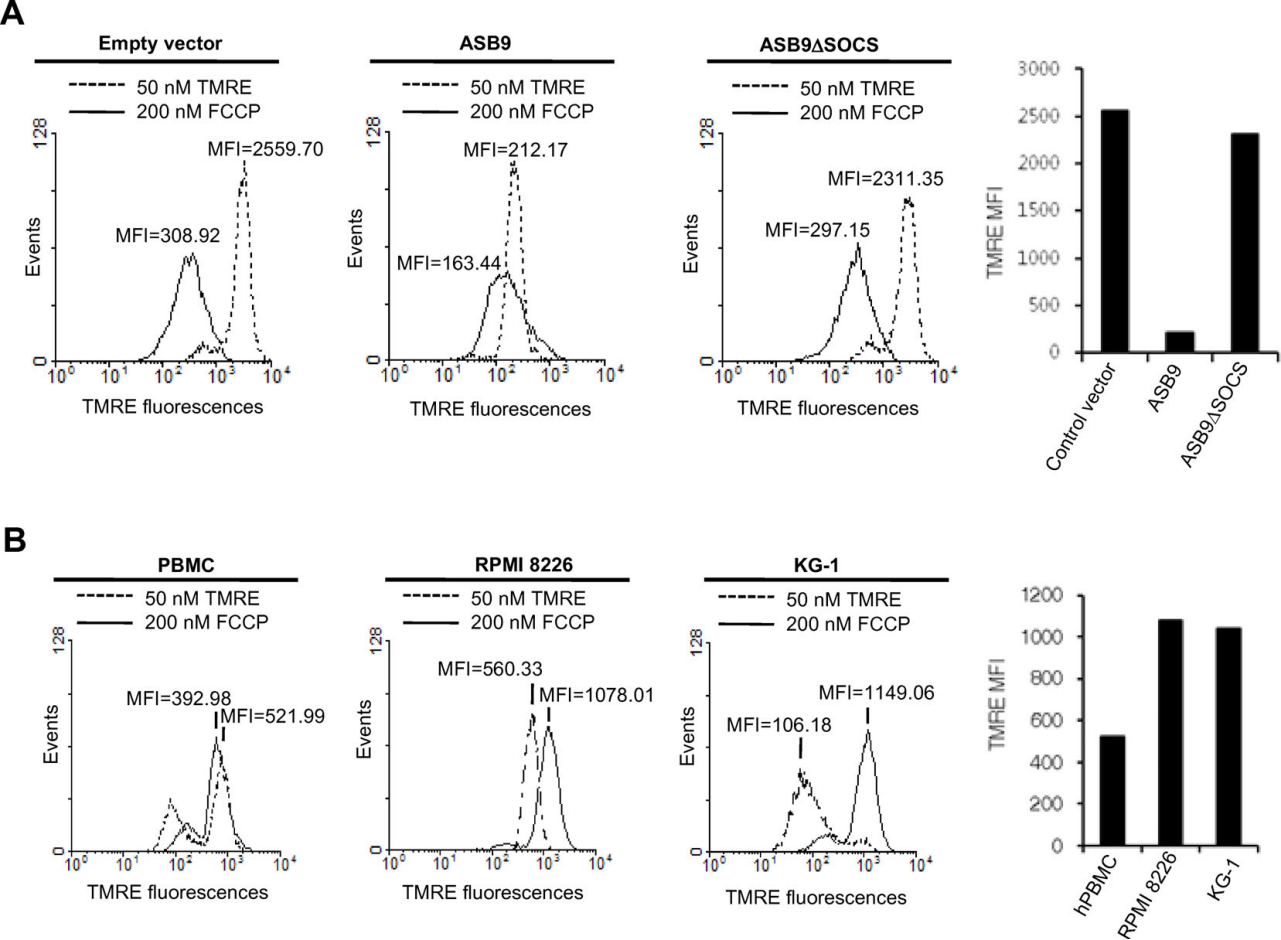
**Effect of human ankyrin repeat and suppressor of cytokine signalling (SOCS) box protein 9 (ASB9) on the mitochondrial membrane potential (ΔΨm)**. **(a) **Human embryonic kidney 293 (HEK293) cells that stably express null (empty vector), Myc-tagged ASB9, or ASB9ΔSOCS were trypsinised for 5 min and incubated in 50 nM tetramethylrhodamine ethyl ester (TMRE) with (full line) or without (dotted line) 200 nM carbonyl cyanide p-(trifluoromethoxy) phenylhydrazone (FCCP) for 30 min at 37°C in the dark. The TMRE fluorescence in the cells was analysed by flow cytometry. The low fluorescence indicates a loss of ΔΨm. **(b) **The ΔΨm of peripheral blood mononuclear cells (PBMCs), RPMI 8226 cells, and KG-1 cells were analysed as described in (a).

To directly investigate the correlation of ASB9-induced change in the mitochondrial structure with mitochondrial function, we used tetramethylrhodamine ethyl ester (TMRE) to analyse the mitochondrial membrane potential (ΔΨm). TMRE is a highly fluorescent cationic lipophilic dye used to determine the value of ΔΨm based on the fluorescence intensity [27]. The membrane potential ΔΨm was detected in the control HEK293 cells and shifted leftward in the presence of the uncoupler, carbonyl cyanide *p*-(trifluoromethoxy) phenylhydrazone (FCCP), indicating the depolarisation of ΔΨm (Figure 13a, left panel). The ΔΨm in the cells that stably express ASB9 was significantly lower than that of the control cells, which is similar to the membrane potential in the presence of the uncoupler, indicating the dissipation of ΔΨm in the ASB9 expressing cells (Figure 13a, middle panel). In the ASB9ΔSOCS expressing cells, the membrane potential was similar to that of the control cells (Figure 13a, right panel). This result suggests that the ubiquitination and degradation of uMtCK in ASB9-expressing cells is associated with a change in the mitochondrial structure and ΔΨm, which results in a significantly impaired mitochondrial function that may change a variety of cellular functions. To further confirm the correlation of ASB9 expression and ΔΨm, we analysed ΔΨm in the PBMC, RPMI 8226 cells and KG-1 cells (Figure 13b). The ΔΨm in the PBMC (expressing ASB9 but not ASB9ΔSOCS) was significantly lower than that of the RPMI 8226 cells and KG-1 cells. The dissipation of ΔΨm in the presence of the uncoupler was hardly detected in the PBMC. These results potentially support that the regulation of ASB9 and ASB9ΔSOCS expression may be a critical factor for the different capability of growth in primary cells and cell lines.

### Effect of ASB9 on CK activity

**Figure 14:**
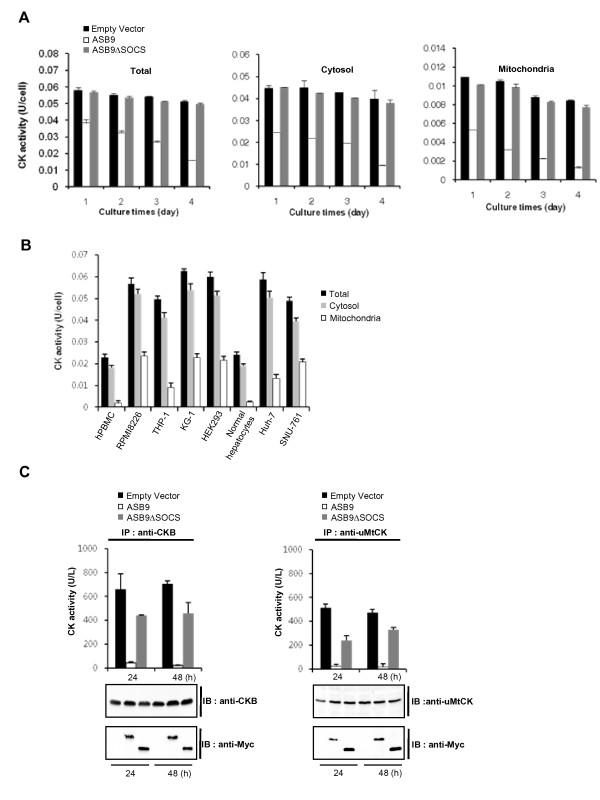
**Effect of human ankyrin repeat and suppressor of cytokine signalling (SOCS) box protein 9 (ASB9) on creatine kinase (CK) activity**. **(a) **The cellular CK activity was measured during a 4-day culture of the human embryonic kidney 293 (HEK293) cells that stably express null, Myc-tagged ASB9, or ASB9ΔSOCS. The cell lysates (total cells), cytosolic fraction and mitochondrial fraction were prepared, and the CK activity was determined. **(b) **The cell lysates (total cells), cytosolic fraction and mitochondrial fraction of human peripheral blood mononuclear cells (hPBMCs), normal hepatocytes and human cell lines were prepared, and the CK activity was determined. **(c) **The immunocomplex proteins of the anti-CKB or anti-ubiquitous mitochondrial CK (uMtCK) antibodies were analysed by CK activity assay. Specific CK activity was defined in terms of units per cell (U/cell). The immunoprecipitated proteins were detected by immunoblotting analysis with an anti-CKB antibody and an anti-Myc antibody (for ASB9) (lower panel).

The CKB in the cytosol and the uMtCK in the mitochondria interact with ASB9 and are degraded by ASB9 (Figures 4, 5, 6, 7, 8, 9, 10, 11). Thus, the correlation of CK activity with the ASB9 expression was investigated in the HEK293 cells that stably express ASB9 or ASB9ΔSOCS (Figure 14). The total cellular CK activity was reduced only in the HEK293 cells that express ASB9 but not ASB9ΔSOCS. Next, the CK activity was compared in the cytosol and mitochondrial fractions. The cytosolic and mitochondrial types of CK activity were both reduced in the ASB9-expressing cells (Figure 14a). However, there was no significant reduction in the CK activity in the cells that express ASB9ΔSOCS. To confirm the correlation of CK activity with the ASB9 expression, we analysed CK activity from human cell lines, PBMCs and normal hepatocytes. The total cellular, cytosolic and mitochondrial types of CK activity were significantly reduced in the PBMCs and normal hepatocytes that express ASB9 but not ASB9ΔSOCS (Figure 14b). Furthermore, the mitochondrial type of CK activity in the THP-1 and Huh-7 cells (expression of low level uMtCK, Figure 3c) was lower than that of the other human cell lines (Figure 14b). To confirm the activity of CKB and uMtCK more accurately, these proteins were immunoprecipitated with anti-CKB and anti-uMtCK, and the activity of the immunocomplexes was measured. As shown in Figure 14c, ASB9 clearly abolished the CK activity of CKB and uMtCK. The CK activity of uMtCK was also decreased in the presence of ASB9ΔSOCS. These results imply that the association of ASB9ΔSOCS with uMtCK may reduce the enzymatic activity even though uMtCK is not ubiquitinated and degraded. They also confirm that the involvement of ASB9 in the creatine kinase system depends on the SOCS box.

### Effect of ASB9 on cell growth

**Figure 15:**
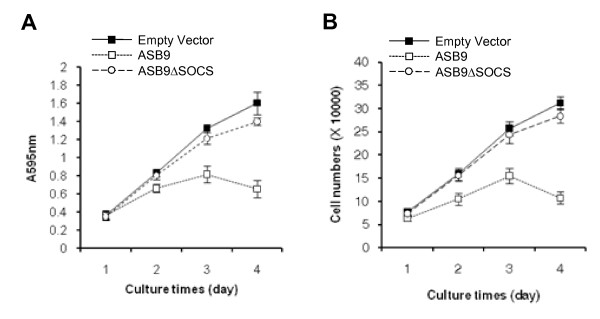
**Effect of human ankyrin repeat and suppressor of cytokine signalling (SOCS) box protein 9 (ASB9) on cell growth**. The cell growth was measured during a 4-day culture of the human embryonic kidney 293 (HEK293) cells that stably express null, Myc-tagged ASB9, or ASB9ΔSOCS. Cell growth was measured by means of a 3-(4,5-dimethylthiazol-2-yl)-2,5-diphenyltetrazolium bromide (MTT) assay **(a) **and a trypan blue exclusion assay **(b)**.

To investigate the correlation of ASB9 with cell growth, we performed a 3-(4,5-dimethylthiazol-2-yl)-2,5-diphenyltetrazolium bromide (MTT) assay on the HEK293 cells that stably express ASB9 or ASB9ΔSOCS. The cell growth was markedly delayed in the cells that express ASB9 but not ASB9ΔSOCS (Figure 15a). Given that the MTT assay measures the mitochondrial dehydrogenase enzyme activity [28], we deduce from the results that ASB9 is directly involved in the mitochondrial function and cell growth. Additionally, we obtained the same results when we checked the cell growth by using the trypan blue exclusion method (Figure 15b).

## Discussion

Because Asbs interact with a wide variety of target substrates via ankyrin repeat domains, they have diverse functions such as regulation of proliferation, differentiation, carcinogenesis, and regulation of the cell cycle. Therefore, ASBs may be differentially expressed between cell lines and primary cells. Here, we evaluated expression and function of ASB9 in detail. When investigating the expression of ASBs, we detected a splicing variant of the ASB9 that lacks a SOCS box (ASB9ΔSOCS) in several cell lines. Recently, ASB9 was reported to interact with CKB in HEK293 cells, indicating that ASB9 mediates ubiquitination and proteasomal degradation of CKB in the cells. The investigators artificially deleted the SOCS box by using recombinant biotechnology and proved that the SOCS box of ASB9 mediates interactions with the elongin B/elongin C/Cullin 5 complex by functioning as an adaptor for an E3 ubiquitin ligase complex [23]. Here, we noticed that ASB9ΔSOCS is naturally present in human cell lines. Interestingly, ASB9ΔSOCS is not expressed in the PBMCs from diverse blood samples and normal hepatocytes (Figures 1 and 3). In the process of cell line establishment, chromosomal rearrangements, insertions, and/or deletions are commonly observed. Given the importance of the SOCS box in ASB9, it is possible that induction of ASB9ΔSOCS by genetic alterations may inhibit normal function of ASB9 and have implications in the regulation of target proteins and cell growth. Recently, ASB9 was isolated as a potential biomarker for breast cancer [24]. It will be interesting to investigate whether the reported increase of ASB9 expression is derived from upregulation of ASB9ΔSOCS rather than ASB9 in breast cancer cells.

On the basis of the proteomics approach, we confirmed that ASB9 interacts with CKs and ubiquitin ligase complexes in the HEK293 cells that stably express Myc-tagged ASB9 (Figures 4 and 5). The peptides identified from the ASB9 interacting proteins covered 27% and 4% of the entire CKB and uMtCK proteins, respectively (Figure 4 and Table 1). In this study, we identify for the first time that uMtCK is a new target of ASB9, in addition to CKB. Because the co-operative involvement of cytosolic CKs and mitochondrial CKs is required for a functional phosphocreatine circuit [10-14], it is ideal that ASB9 recruits and regulates CKB in cytosol and uMtCK in mitochondria. According to the transfection and immunoprecipitation study, the ANK repeat domains of ASB9 can associate with the substrate binding site of uMtCK (Figures 7 and 8). This result is in agreement with previous reports on the binding properties of Asbs and CKs. Furthermore, the expression of ASB9 induces the ubiquitination of uMtCK and CKB *in vivo *and *in vitro *(Figures 9 and 10). As a result, the protein levels of uMtCK and CKB were clearly reduced after long-term culture for 10 passages (Figure 11).

Given that ASB9 interacts with uMtCK, we reasoned that the two proteins colocalise in the mitochondria. ASB9 or ASB9ΔSOCS colocalised with endogenous uMtCK in the mitochondria as expected (Figure 12). Interestingly, the expression of ASB9 induced an abnormal mitochondrial structure and loss of membrane potential. Nowadays, the paradigm of the mitochondrial function is changing: mitochondria are considered not merely simple ATP-generating organelles but complex integrators of cellular signalling pathways [29,30]. Studies that use fluorescence staining in live cells to investigate the structure of mitochondria suggest that mitochondria are highly dynamic [31]. Furthermore, mitochondrial alterations appear to be a factor in cancer [32,33]. Therefore, the abnormal structure of mitochondria (Figure 12) and loss of ΔΨm (Figure 13) may be direct evidence that ASB9 severely interrupts the normal function of mitochondria. Previous studies by many investigators have shown that MtCK proteins form an active octameric complex with an internal channel that stabilises the contact site between the outer membrane and the inner membrane and that this behaviour may be essential for the proper function [13,14]. Moreover, under situations of a compromised cellular energy state, such as ischaemia, oxidative stress, and calcium overload, MtCK seems to be a susceptible target for oxidation; compensatory upregulation of MtCK gene expression has also been reported [18,19]. In addition, the downregulation of uMtCK by small interfering RNA (siRNA) in HaCaT and HeLaS3 cells reportedly affects cell viability and mitochondrial morphology [34]. Therefore, the targeting of ASB9 to uMtCK is quite proper for efficient regulation of cell growth. The association with ASB9 and degradation via ubiquitination may reduce the level of monomeric uMtCK as well as active oligomeric uMtCK and lead to an abnormal mitochondrial structure and function.

The expression of ASB9 was correlated with the enzyme activity of the target substrates CKB and uMtCK (Figure 14). Furthermore, the cellular CK activity of the total, cytosolic, and mitochondrial fractions were all significantly reduced by ASB9 but not by ASB9ΔSOCS. When we measured the activity of CKB and uMtCK after immunoprecipitation with anti-CKB or anti-uMtCK, the results clearly show that the activity of CKB and uMtCK was abolished by ASB9 and decreased by ASB9ΔSOCS. The association of ASB9ΔSOCS with uMtCK is likely to inhibit the enzymatic activity even though uMtCK is not ubiquitinated and degraded. The difference in the CK activity of cell lysates and immunoprecipitates may be derived from the residual adenylate kinase activity in the cell lysates. Note also that the structure of the high-energy phosphate transfer system is reportedly rather complex because adenylate kinase can compensate for the decrease of CK activity when the action of CKs is reduced [35]. The inhibition of CKB and uMtCK activity by ASB9 is also linked to cell growth (Figure 15). However, as with the CK activity in cell lysates, ASB9ΔSOCS did not show any significant inhibition of cell growth. Therefore, the CK activity of cell lysates may represent the real state of the compromised cells that use a cellular buffer system, possibly adenylate kinases, to control the interruption by ASB9ΔSOCS.

## Conclusions

Our results consistently show that ASB9 targets uMtCK in addition to CKB for degradation, leading to negative regulation of the creatine kinase system and cell growth. Co-operative inhibition of CKB and uMtCK in cytosol and mitochondria may be an efficacious strategy used by ASB9 for the regulation of the cellular energy state. ASB9ΔSOCS can associate with CKs but cannot recruit the E3 ligase complex and ubiquitinate CKB and uMtCK. Thus, ASB9ΔSOCS is likely to be a negative regulator against the function of ASB9. Perhaps the regulation of ASB9 and ASB9ΔSOCS expression will be an escaping strategy for cancer cells or established cell lines from the suppressive function of ASB9. Because the physiological meaning of ASB9ΔSOCS in human cell lines and cancer may give insights into cancer biology, further study on the function of ASB9ΔSOCS is warranted.

## Methods

### Antibodies and plasmids

The anti-Myc and anti-ASB9 antibodies were purchased from Cell Signaling Technology (Beverly, MA, USA) and Abnova (Taipei City, Taiwan), respectively; and the anti-CKB, anti-uMtCK, anti-elongin B, and anti-ubiquitin antibodies were obtained from Santa Cruz (Santa Cruz, CA, USA). The anti-HA and anti-Flag antibodies were obtained from Sigma Aldrich (St Louis, MO, USA), while the anti-goat-Alexa fluor 488 and anti-mouse-Alexa fluor 633 were obtained from Molecular Probes (Eugene, OR, USA). The FLAG-tagged ubiquitin expression vector (pCS4-Flag-Ub) was described previously [36]. The pDSRed2-Mito vector for the detection of mitochondria was purchased from Clontech (Mountain View, CA, USA).

### Mice

Mice were maintained under specific-pathogen-free conditions. Male BALB/c mice (4 weeks old) were purchased from Central Lab. Animal Inc. (Seoul, Korea). Our animal studies were approved by the Institutional Animal Care and Use Committee of Hallym University (Hallym 2008-12). The mice were killed and the peritoneal cells, splenocytes and thymocytes were collected.

### Total RNA isolation and reverse-transcription PCR analysis

Total RNAs were extracted with an RNeasy Mini Kit (Qiagen, Germantown, MD, USA), and the cDNA was generated as described previously [37]. The standard PCR reaction was performed for 25 cycles with the following primer sets: human β-actin, 5'-GGGTCAGAAGGATTCCTATG-3' and 5'-CCTTAATGTCACGCACGATTT-3'; CKB, 5'-ATCGACGACCACTTCCTCTT-3' and 5'-ACCAGCTCCACCTCTGAGAA-3'; and uMtCK, 5'-AGCTTATTGATGACCACTTTCT-3' and 5'-GACGCCGTTCACAATCAATC-3'. mouse glyceraldehyde 3-phosphate dehydrogenase (GAPDH), 5'-ATGGTGAAGGTCGGTGTGAACG-3' and 5'-GTTGTCATGGATGATCTTGGCC-3'; mAsb-9, 5'-TTCGGAGCAAACGCTCAAGCC-3' and 5'-CTTCATTCTGCAAGAAGATCTGGAT-3'. To analyse the expression of 18 members of the ASB family, we used the primers as described in Table 2. For quantitative analysis of the tissue distribution of ASB9, we performed a real-time PCR by using the cDNAs from several human tissues (Clontech) and by using the CYBR Green Master Mix with 5'-AAAACCTAAGCCCTAATCTGTTGACA-3' and 5'-AGGACGAGTTTGGTTATCTTATGATG-3' as primers. The samples were run and analysed with the ABI 7000 machine (Applied Biosystems, Foster City, CA, USA).

**Table 2:** RT primer sequences for human ankyrin repeat and suppressor of cytokine signalling (SOCS) box proteins (ASBs)

Gene	Direction	Sequence	Size (bp)
ASB1	Sense	5'-GTATGTGGCTGTGGTGAACG-3'	359
	Antisense	5'-AGCCCCTGTAGAATCCCTGT-3'	
ASB2	Sense	5'-CCAAGAAGGGCAACTACAGG-3'	382
	Antisense	5'-GCGATATAGGCGTCGATGTT-3'	
ASB3	Sense	5'-TTACTAACCGGGCCTGTGAC-3'	395
	Antisense	5'-AACCAGAAGATGTGGCAACC-3'	
ASB4	Sense	5'-CTTGTGAAATGGCCAATGTG-3'	392
	Antisense	5'-TTCGGCTTTGTAGTCAAGCA-3'	
ASB5	Sense	5'-ATGGCGTGACTCCGTTATTC-3'	337
	Antisense	5'-TCTGTGCTGGATTGTTGAGC-3'	
ASB6	Sense	5'-ACCTGCATCATCTTCCTGCT-3'	361
	Antisense	5'-TCACCATGAGATCCAGGACA-3'	
ASB7	Sense	5'-TGGAGTTCAAGGCTGAGGTT-3'	316
	Antisense	5'-GTGTTCGTGTCTGCTCCGTA-3'	
ASB8	Sense	5'-ATGAGGCTTGTGTGGAGGTC-3'	316
	Antisense	5'-TCTGTTCCAAGTCCCCTGAC-3'	
ASB9	Sense	5'-TGGCCCAGCTCTTCTTGGAG-3'	115
	Antisense	5'-AGGACGAGTTTGGTTATCTTATGATG-3'	
ASB10	Sense	5'-GCTCCTGATTCCGTCTTTGA-3'	395
	Antisense	5'-ACTCTCGCTCCAAACCTGAG-3'	
ASB11	Sense	5'-AAGACCTCCAAGGACGAAGC-3'	152
	Antisense	5'-CCAGCAATCTCCCTGAAATG-3'	
ASB12	Sense	5'-ATGTTCTGGCCCCCTCTATT-3'	326
	Antisense	5'-GGACGACTAAACGGACCTGT-3'	
ASB13	Sense	5'-CCGAATGTGTGAGGCTTCTT-3'	375
	Antisense	5'-CAGTGGCCTTCCTCAAGTTC-3'	
ASB14	Sense	5'-CAAGCTGCTTCTGAGTGCTG-3'	557
	Antisense	5'-GAGAGATGTTGCAGCCATGA-3'	
ASB15	Sense	5'-AATGCAATTCGGAAAAGTGG-3'	363
	Antisense	5'-AATGACACTGGGGAAACGAG-3'	
ASB16	Sense	5'-AGACTGTGCTCGACACCTGA-3'	117
	Antisense	5'-CAGAGGTGCAAAGGAGTCGT-3'	
ASB17	Sense	5'-GGGTTTTTGCCAGAAAAGGT-3'	307
	Antisense	5'-TCAGCCAATTCACGATCAAC-3'	
ASB18	Sense	5'-GATGAGATGGAATGGCAGGT-3'	312
	Antisense	5'-GGGACAGATGCACAGGTCTT-3'	

### Construction of expression plasmids for recombinant ASB9 and uMtCK

The ASB9 and ASB9ΔSOCS cDNAs were amplified from HEK293 cells by RT-PCR using the following primer sets: ASB9 and ASB9ΔSOCS 5' primer, 5'-CTCGAGATGGATGGCAAACAAGGGGG-3'; ASB9 3'primer, 5'-GGTACCAGATGTAGGAGAAACTGTTTC-3'; and ASB9ΔSOCS 3' primer, 5'-TGGTACCGGCTTAGGTTTTGGCAAAG-3'. The nucleotide sequences of the cDNAs were verified by DNA sequencing and a BLAST search. The ASB9 ANK repeat deletion mutants were generated by PCR using the following primers in combination with ASB9 3' primer: ASB9ΔANK1 5'-primer, 5'-CTCGAGATGGATCATGTTTCCCCACTCC-3'; ASB9ΔANK1-2 5'-primer, 5'-CTCGAGATGGACTGGCACACTCCACTG-3'; ASB9ΔANK1-3 5'-primer, 5'-CTCGAGATGCTGGCATCCCCCATCCAT-3'; and ASB9ΔANK1-5 5'-primer 5'-CTCGAGATGCAGGATTCCCCACTTCATG-3'. The uMtCK cDNA from HEK293 cells was amplified by RT-PCR with 5'-primer, 5'-GGATCCAAATGGCTGGTCCCTTCTC-3' and 3'-primer, 5'-AAGCTTTATAGGGAGGCTAGCTATCTA-3'. To delete the BS of uMtCK (amino acids 135 to 145), we used the two primer sets: one for the partial uMtCK sequence covering downstream of BS, 5'-GGTACCGTGGTGGATGCACTGAGTG-3' and uMtCK 3' primer; and one for the partial uMtCK sequence encoding upstream of BS, uMtCK 5' primer and 5'-TTGCTGACCTGTTTGACCCTGGTACC-3'. The two partial sequences were ligated by using the *Kpn*I restriction enzyme site. The cDNA fragments were cloned into the mammalian expression vectors pcDNA-3.1/Myc-His(-)B (Invitrogen, Carlsbad, CA, USA) (ASB9, ASB9ΔSOCS and ASB9ΔANK) and pcDNA-3.1/HA (uMtCK and uMtCKΔBS).

### Cell culture and construction of stable cell lines

Human keratinocyte (HaCaT) cells were kindly provided by Professor N Fusenig (German Cancer Research Center, Heidelberg, Germany). The human embryonic kidney cell (HEK293), human B cell (RPMI 8226), human acute monocytic leukaemia cell (THP-1), human myeloid cell (KG-1), human hepatoma liver carcinoma cell (HepG-2) and human hepatoma cell (Huh-7) lines were obtained from the American Type Culture Collection (ATCC, Manassas, VA, USA). The human hepatocellular cell lines (SNU-398, SNU-423, SNU-739 and SNU-761) were from the Korean Cell Line Bank (Seoul, Korea). The HaCaT and HEK293 cells were maintained in Dulbecco's modified Eagle medium (DMEM) with 10% fetal bovine serum (FBS; Hyclone, Logan, UT, USA). The RPMI 8226, THP-1, HepG-2, Huh-7, SNU-398, SNU-423, SNU-739 and SNU-761 cells were maintained in an RPMI 1640 medium with 10% FBS, and the KG-1 cells were maintained in Iscove's modified Dulbecco's media (IMDM) with 20% FBS. The human normal hepatocyte cell (Promo Cell, Heidelberg, Germany) was maintained as the vendor suggested with Institutional Review Board approval. All cells were cultured at 37°C in an atmosphere of 95% air and 5% CO_2_. For the generation of ASB9 and ASB9ΔSOCS-expressing stable cell lines, the HEK293 cells were transfected with FuGENE 6 (Roche, Indianapolis, IN, USA) and the cells were selected in 500 μg/ml G418 (Duchefa Biochemie, Haarlem, The Netherlands) for 14 days. The expression of Myc-tagged ASB9 and ASB9ΔSOCS was confirmed by means of immunoblotting with the anti-Myc antibody.

### Immunoprecipitation

The cells were lysed for 30 min at 4°C in an immunoprecipitation (IP) buffer (10 mM 4-(2-hydroxyethyl)-1-piperazineethanesulfonic acid (HEPES), pH 7.4, 150 mM NaCl, 5 mM ethylenediaminetetraacetic acid (EDTA), complete protease inhibitor cocktail (Roche), 100 mM NaF and 2 mM Na_3_VO_4_) supplemented with 1.0% nonyl phenoxylpolyethoxylethanol (NP-40). Cell debris was removed by centrifugation, and cell lysates were incubated with the indicated antibodies for 2 h at 4°C. Protein A-Sepharose CL-4B (10% (v/v) slurry, Amersham Pharmacia Biotech, Piscataway, NJ, USA) was added, and the reaction mixtures were subjected to an additional 2 h of incubation at 4°C. The immunocomplexes collected by centrifugation were washed twice with an IP buffer that contained 0.1% NP-40.

### Protein identification by mass spectrometry

Immunocomplexes were resolved by 15% SDS-PAGE and stained with Coomassie brilliant blue G-250. For identification of ASB9 and its binding proteins, the protein bands were digested in gel with trypsin and the resulting peptides were then purified with a POROS R2 column (Applied Biosystems) to concentrate and desalt the sample. The samples were analysed by electrospray ionisation-time of flight mass spectrometry/mass spectrometry (ESI-TOF MS/MS) using a Micromass Q-TOF MA equipped with a nanospray source (Waters, Milford, MA, USA) in an In2 gen facility (Seoul, Republic of Korea). The resulting amino acid sequences were further analysed using the database managed by the National Center for Biotechnology Information http://www.ncbi.nlm.nih.gov.

### Immunoblotting analysis

The immunocomplexes and cell lysates were resolved by 12.5% SDS-PAGE. The separated proteins were transferred to a nitrocellulose membrane (Bio-Rad, Hercules, CA, USA), blocked in PBS containing 0.05% Tween-20 and 3% skim milk for 1 h, and incubated with the indicated antibodies for 2 h at room temperature. Immunoreactive proteins were detected by means of a horseradish peroxidase-conjugated secondary antibody (Cell Signaling Technology) and an enhanced chemiluminescence reagent (Amersham Pharmacia Biotech).

### *In vitro *ubiquitination assay

Immunocomplexes obtained by anti-CKB or anti-uMtCK antibodies were mixed with 50 μl of a reaction buffer containing 20 mM HEPES (pH 7.3), 10 mM MgCl_2_, 2 mM ATP, 1 mM dithiothreitol, 5 μg/ml Ub, 50 μM Ub-activating enzyme (E1), and 1 μM ubiquitin-conjugating enzyme (E2-UbcH5a) (Boston Biochem, Cambridge, MA, USA) and incubated for 1.5 h at 37°C. Polyubiquitination was identified by SDS-PAGE and immunoblotting analysis with the anti-ubiquitin antibody.

### Immunofluorescence staining and confocal microscopy

The cells were cultured on glass coverslips in 12-well plates for 24 h and transfected with a pDSRed2-Mito vector (Clontech) for the detection of mitochondria. After 24 h, cells were fixed with 4% paraformaldehyde, permeabilised with 0.1% Triton X-100, incubated with antibodies against Myc epitope (for ASB9) and uMtCK, and then incubated with Alexa fluor 633 conjugated goat anti-mouse IgG and Alexa fluor 488 conjugated donkey anti-goat IgG (Molecular Probes). Nuclei were stained with Hoechst no. 33258. The mounted samples were scanned with an LSM 510 microscope (Carl Zeiss, Jena, Germany). For three-dimensional analysis of mitochondrial structure in control cells and ASB9 overexpressing cells, we acquired Z-scan images with a Z-slice interval 0.34 μM. A three-dimensional animation file was converted with angle degree 6° using LSM510 META software (Carl Zeiss) for three-dimensional reconstruction (see Additional files 1 and 2).

### Analysis of mitochondrial membrane potential

To measure the ΔΨm, we incubated the cells with 50 nM TMRE (Sigma Aldrich) for 30 min at 37°C. FCCP (Molecular Probes) was treated for 30 min at 37°C as an uncoupler. The cells were analysed with a FACScan flow cytometer (BD Biosciences, San Jose, CA, USA) to monitor the TMRE fluorescence (582 nm, FL-2 channel).

### Mitochondrial fractionation

The mitochondrial fractions were isolated using a mitochondrial fractionation kit (Active Motif, Carlsbad, CA, USA) in accordance with the manufacturer's specifications. After the cells were washed and homogenised, the cell debris was removed by centrifugation at 800 *g *for 20 min at 4°C; the supernatants were then centrifuged at 10,000 *g *for 20 min at 4°C to separate the mitochondrial (pellets) and the cytosolic fraction (supernatants).

### CK activity assay

The CK activity was determined with the EnzyChrom Creatine Kinase Assay Kit (BioAssay Systems, Hayward, CA, USA) in accordance with the manufacturer's specifications. The colour development was measured at 340 nm. The specific CK activity was defined in terms of units per cell (U/cell).

### Cell viability assay

The cell growth of the HEK293 cells was determined by an MTT assay with a MTT solution (Sigma Aldrich) as described previously [38]. For the MTT assay, the cells were seeded in 48-well plates. After the indicated culture periods, the MTT solution was added to each well and the plates were incubated for an additional 4 h at 37°C. After removal of the medium, the formazan crystals were solubilised in dumethylsufloxide (DMSO). The colour development was monitored by a spectrophotometer at 595 nm with a reference wavelength of 650 nm. The number of viable cells was counted by means of the trypan blue dye exclusion method.

## Authors' contributions

HJK and YL designed the research, supervised the experiments and wrote the paper; SK and DK performed the molecular research, analysed the function of ASB9 and prepared figures; JWR and JAP performed flow cytometry experiments and analysed data; DWK and DSK contributed to *in vitro *ubiquitination assay experiments and participated in paper writing; all authors read and approved the final manuscript.

## Supplementary Material

Additional file 1 **Movie 1**. For three-dimensional analysis of mitochondrial structure in control cells, we acquired Z-scan image with Z-slice interval 0.34 μM. The three-dimensional animation file was converted with angle degree 6° using LSM510 META software for three-dimensional reconstruction. Click here for file

Additional file 2 **Movie 2**. For three-dimensional analysis of the mitochondrial structure of human ankyrin repeat and suppressor of cytokine signalling (SOCS) box protein 9 (ASB9) (movie 2) overexpressing cells (see Figure 12b), we acquired Z-scan image with Z-slice interval 0.34 μM. The three-dimensional animation file was converted with angle degree 6° using LSM510 META software for three-dimensional reconstruction. Click here for file
